# A Review and Comparison of Methods for Recreating Individual Patient Data from Published Kaplan-Meier Survival Curves for Economic Evaluations: A Simulation Study

**DOI:** 10.1371/journal.pone.0121353

**Published:** 2015-03-24

**Authors:** Xiaomin Wan, Liubao Peng, Yuanjian Li

**Affiliations:** 1 Department of Pharmacy, the Second Xiangya Hospital, Central South University, Changsha 410011, China; 2 College of Pharmacy, Central South University, Changsha 410083, China; Cardiff University, UNITED KINGDOM

## Abstract

**Background:**

In general, the individual patient-level data (IPD) collected in clinical trials are not available to independent researchers to conduct economic evaluations; researchers only have access to published survival curves and summary statistics. Thus, methods that use published survival curves and summary statistics to reproduce statistics for economic evaluations are essential. Four methods have been identified: two traditional methods 1) least squares method, 2) graphical method; and two recently proposed methods by 3) Hoyle and Henley, 4) Guyot et al. The four methods were first individually reviewed and subsequently assessed regarding their abilities to estimate mean survival through a simulation study.

**Methods:**

A number of different scenarios were developed that comprised combinations of various sample sizes, censoring rates and parametric survival distributions. One thousand simulated survival datasets were generated for each scenario, and all methods were applied to actual IPD. The uncertainty in the estimate of mean survival time was also captured.

**Results:**

All methods provided accurate estimates of the mean survival time when the sample size was 500 and a Weibull distribution was used. When the sample size was 100 and the Weibull distribution was used, the Guyot et al. method was almost as accurate as the Hoyle and Henley method; however, more biases were identified in the traditional methods. When a lognormal distribution was used, the Guyot et al. method generated noticeably less bias and a more accurate uncertainty compared with the Hoyle and Henley method.

**Conclusions:**

The traditional methods should not be preferred because of their remarkable overestimation. When the Weibull distribution was used for a fitted model, the Guyot et al. method was almost as accurate as the Hoyle and Henley method. However, if the lognormal distribution was used, the Guyot et al. method was less biased compared with the Hoyle and Henley method.

## Introduction

Decision analytic modeling is often applied as a vehicle for economic evaluations. [1] A commonly used approach in decision analytic modeling is the Markov model. Transition probabilities between Markov states are one of the most important features of the stochastic model. There are several available methods for the estimation of transition probabilities. Currently, survival analysis has been frequently used to estimate transition probabilities. Recently, an algorithm was developed by Latimer that aimed to improve the quality of survival analyses included within economic evaluations. [2] The framework proposed by Latimer was founded on the assumption that the researchers who conduct economic evaluations have access to individual patient-level data (IPD). Typically, IPD originate from clinical trials and observational studies. However, censoring frequently occurs during the follow-up period, and the key feature of the survival analysis is its ability to facilitate censoring inclusion, which makes it an appropriate method for survival data with censoring.

When IPD are available, the relationship between transition probabilities and time can be estimated from IPD using a survival analysis. The commonly used approach is to fit parametric survival functions to IPD by applying the maximum likelihood estimation (MLE) to estimate the parameters of the distributions that are used for the estimation of the transition probabilities. An estimate of the mean survival time is essential for cost-effectiveness analyses (CEAs); the mean survival time is a measure of the effectiveness in a CEA and can be estimated from parametric survival functions. [3] In addition to the mean survival time, other parameters can also be estimated from survival distributions. The uncertainty of parameters can be captured with a variance-covariance matrix from parametric regression models. [1]

However, IPD are often only available to clinical trial sponsors because of confidentiality, and the independent economic evaluation researchers lack access to these data. Independent researchers can typically obtain published Kaplan–Meier curves and summary statistics, e.g., numbers at risk and total number of events, for economic evaluations.

For these reasons, methods that use published survival curves and reported summary statistics to reproduce statistics for economic evaluations are essential for independent researchers to conduct these evaluations. However, there are few methods available to attain information for economic evaluations in the absence of IPD, and only four methods have been identified to recreate IPD for economic evaluations from published KM curves. These four methods can be used to estimate the parameters of the fitted parametric survival function. The general equation of transition probabilities is as follows: *tp*(*t_u_*) = 1 − exp{*H*(*t* − *u*) − *H*(*t*)}, where *u* indicates the length of the Markov cycle, *t* indicates the survival time, *tp*(*t_u_*) indicates the transition probability between time point t-u and t, and *H(t)* indicates the cumulative hazard function of the parametric distribution. [4] In the following sections, the four identified methods were described briefly and then assessed by using a simulation study.

## Methods

### Method 1: Least squares method

The least squares method involves the estimation of parameters by minimizing the sum of the squares of the residuals; the residuals represent the differences between the actual data and the estimated data from a model. The least squares method is a common method of statistical estimation and is included in almost every statistical package, e.g., the R package, which makes it accessible to researchers in experimental sciences.

Suppose 20 data points, (x_1,_ y_1_), (x_2,_ y_2_),…, (x_20_,y_20_), were extracted from a survival curve that followed a distribution with a function of y = f(x, β), in which β = (β1, β2). The β vectors of the parameters were estimated using the least squares method, which minimizes the sum of squares.

### Method 2: Graphical method

The graphical method used to estimate the parameters of parametric models involves transforming the survivor function to a linear function and fitting a straight line through a series of points that were extracted from a published Kaplan-Meier survival curve [5]. For example, the survival function of the Weibull distribution is provided as follows: $S(t)=e^{-\lambda t^{\gamma }}$. We can transform this distribution by taking the logarithm as follows: log(*S*(*t*)) = − *λt_γ_*, where *λ* is a scale parameter and γ is a shape parameter. We can subsequently take the logarithm again: log(− log(*S*(*t*))) = log(*γ*) + γ*log(*t*). From this equation, we can plot log(log(*S*(*t*))) versus log(*t*), and the parameters can then be estimated. The graphical method typically provides rough estimates of the parameters, but it has been used because of its simplicity.

### Method 3: Estimation of interval-censored data (Hoyle and Henley, 2011)

Hoyle and Henley [6] proposed a method for the estimation of IPD. Numbers at risk, the total number of patients and survival probabilities were used to estimate IPD. The method made an assumption that censoring is constant within each time interval. The number of events and censorships in each time interval of 1/4 length were estimated, which improved the curve fits considerably. When numbers of patients at risk are not reported, the numbers of events and censorship can be estimated by the method proposed by Tierney et al. [7] The method will be more accurate with more reported intervals. The estimated IPD according to this method are interval-censored. A spreadsheet that implements the method is available at http://medicine.exeter.ac.uk/media/universityofexeter/medicalschool/profiles/Hoyle_and_Henley_Version_1.1.xls…

Hoyle and Henley conducted a comparison of their method with two transitional methods, e.g., the least squares and graphical methods, using the Monte Carlo method for comparison. The parameters used in the scenarios in their simulation included sample size, combinations of parameters of the Weibull distribution and censoring type. The results of the simulation demonstrated that the method proposed by Hoyle and Henley was more accurate in both fitting the curve and estimating the mean survival time compared with the traditional methods. Furthermore, the estimation of the mean survival time using the proposed method was as accurate as the estimation obtained by applying the MLE directly to actual IPD. One important feature of this proposed method that distinguishes it from traditional methods is that uncertainty can be captured with this method.

However, alternative survival models and variations in the proportion of censoring were not included in the scenarios in the simulation study. The authors only used the Weibull distribution in their simulation study; however, many candidate survival models exist, such as Weibull, gamma, Gompertz, log-normal, and log-logistic. The lognormal distribution has typically been used to estimate the mean survival in NICE technology appraisals (TAs) instead of the Weibull distribution. [2] The lognormal distribution has commonly been used in the medical field, particularly for fitting cancer site data. [8] In a realistic trial setting, the censoring rate is often very high. [9] For example, a censoring rate as high as 70% is often identified in real clinical data. The MLE method is known to provide biased estimates when the data are heavily censored. [10]

### Method 4: Estimation of precise survival data: survival data with left or right censoring (Guyot et al, 2012)

In 2012, an iterative algorithm developed by Guyot et al. was designed to obtain the reproduced IPD; consequently, survival curves could be reconstructed with the recreated IPD. [11]. The estimated IPD by the method was precise survival data rather than interval censored data. It was assumed that censoring rate is constant within time interval. An initial estimates for the number censored on interval i were obtained. Given this initial estimates, the number censored between extracted KM co-ordinates k and k + 1 was calculated. Then the number of events at each extracted KM co-ordinate and number of patients at risk at the next co-ordinate can be estimated. If the estimated number at risk at the beginning of interval i not equaled to the number at risk reported at the start of i, the process was repeated until the two figures matched from the previous iteration. If the reported total number of events was more than the estimated, the process was repeated until the two figures matched from the previous iteration. Further explanation of the method can be found in the original paper.[11]

Regarding reproducibility and accuracy, the simulation was not performed to test the accuracy of the proposed method, which was different from the method created by Hoyle. Six pairs of survival curves were created from four published articles. The authors assessed the accuracy of the proposed method by comparing the actual 22 survival probabilities, 7 median survival times, 6 hazard ratios and 4 standard errors of the log hazard ratios that were reported in the four publications with the variables reconstructed using the proposed method. The results demonstrated that the accuracy was great for survival probabilities and median survival times, and the accuracy was reasonable when the number of individuals at risk or the total number of events was reported.

However, because the method proposed by Guyot et al. was not compared with other traditional methods, it remains unknown whether the accuracy of the proposed method is greater than the other methods previously described.

Because the Monte Carlo simulation was not performed to test the accuracy of the proposed method, we were not able to model different combinations of values for different inputs to observe the effects of truly different scenarios.

There are similarities and differences in the methods proposed by Hoyle and Henley and Guyot et al. Both methods include the use of the survival curve and the number at risk, as well as the assumption that censoring occurs at a constant rate in each time interval for both methods. The differences are summarized in Table 1.

**Table 1:** Differences between the method by Hoyle and Henley and that by Guyot et al.

Method by Guyot et al.	Method by Hoyle and Henley
Precise IPD was estimated	Interval-censored IPD was estimated
Total number of events was used (if available)	No such data was used
Iterative algorithm was used	Closed-form was used
No comparison with traditional methods	Comparison with traditional methods was made
All computing was done in R	Most parts of computing was done in Excel and some in R
More data preparation work required	Less data preparation work required

Taylor et al. [12] reviewed the non-linear, Guyot et al. and Hoyle and Henley methods and assessed their impacts on the estimates of survival parameters by extracting data points from published survival curves and applying the three methods to the two case studies obtained. Because IPD were unavailable, the accuracy of these three methods was not compared. The findings indicated the methods used to estimate survival parameters to obtain transition probabilities between transition states can affect cost-effectiveness results.

Although the accuracy between Hoyle and Henley’s method and traditional methods has been compared, this comparison was performed in the absence of the method proposed by Guyot et al., which is an important method to help researchers recreate survival data that have been cited and used frequently. [2, 4, 13–15] Additionally, it is unclear how the four methods perform in different situations in which alternative survival models and various censoring rates are assumed to reflect realistic clinical data. Therefore, we evaluated the four methods using a simulation study.

### Simulation settings

To compare the four methods previously described, a simulation study that could generate survival data with censoring was designed and conducted. This study enabled us to compare the performance of the four methods. The simulated survival data were designed to reflect real clinical trial data. This section contains the details of the simulation study design. The simulation settings in this study are similar to Hoyle and Henley’s study, with the exceptions that (1) the Guyot et al. method was included, (2) variations in the proportion of censoring were simulated, and (3) the lognormal distribution was used in addition to the Weibull distribution.

### Survival times

To simulate data, a number of patients with an underling survival time must be generated. Two sample sizes were chosen: 100 and 500 individuals. These sample sizes reflect the sample sizes typically included in clinical trials. [16–18] To simulate survival times with censoring, two survival distributions were required, one distribution for the time-to-event (T_i_) and a second distribution for time to censoring (C_i_). The Weibull and lognormal distributions are most commonly used for time-to-event data. [19] In this simulation, we considered these two survival distributions for the uncensored survival time T_i_: (1) Weibull distribution; and (2) lognormal distribution.

The survival function of the Weibull distribution is *S*(*t*) = exp(− *λt^γ^*), in which *λ* is a scale parameter and *γ* is a shape parameter. The mean survival time of the Weibull distribution can be estimated as follows: *E*(X) = *λ*Γ(1 + 1/*γ*), in which Γ is the gamma function. In the simulations, the mean survival time was set to 10 time units, which is common in time-to-event data. To ensure the mean survival time remained at 10 units, different combinations of the scale and shape parameters were chosen. First, three shape parameters were chosen that corresponded to the parameters used by Hoyle and Henley; these parameters cover most situations that would be encountered in the Weibull distribution. Second, the scale parameter was set to make the mean survival time equal to 10 units. The combinations of the parameters were as follows:

1. Decreasing failure rate (scale parameter<1): γ = 0.6, λ = 6.65
2. Constant failure rate (scale parameter<1): γ = 1, λ = 10
3. Increasing failure rate (scale parameter>1): λ = 2, λ = 11.28.

The survival function of the lognormal distribution is $S(t)=1-\Phi (\frac{\ln(t)-u}{\sigma })$, in which Φ is the cumulative distribution function of the normal distribution,$\Phi \left(x\right)=\int_{-\infty }^{x}\frac{e^{-x^{2}/2}}{\sqrt{2\pi }}$, and μ and σ are the mean and standard deviation, respectively, of the variable’s natural logarithm. The mean survival time of the lognormal distribution can be expressed as:$E\left(X\right)=e^{\mu +\frac{1}{2}\sigma^{2}}$. The mean survival time of the lognormal distribution was also set to 10 units.

The hazard function of the lognormal distribution first increases from zero to a maximum value and then decreases back to zero. The amount that the hazard function increases or decreases primarily depends on the value of *σ*.

The combinations of the parameters of the lognormal distribution were as follows:

1. σ = 1, μ = 1.802585: The hazard rate first increases from zero to a maximum value and then decreases to zero;
2. σ = 2, μ = 0.3025851: The hazard function essentially decreases over most time values.

The type of censoring in the simulation included type I censoring (because of pre-assigned fixed censoring times) and random censoring (because of loss to follow-up). The common censoring distributions considered in the literature are typically uniform and have exponential distributions [20]; in the simulation study, the random censoring was generated from an exponential distribution. The mean values of the exponential distributions were set to attain the desired censoring rates of 26, 42, and 76% to simulate what occurs in the real world (Table 2).

**Table 2:** The mean of the exponential distribution in simulations.

Censoring rate	Distribution				
	Weibull (γ,λ)			Lognormal(σ, μ)	
	(0.6, 6.65)	(1, 10)	(2, 11.28)	(1, 1.802585)	(2, 0.3025851)
26%	a	b	b	c	13
42%	10	33	200	20	4
76%	1	3.5	7	3.5	0.5

a: The mean of exponential distribution was set to a large number(e.g. 10000000) in order to attain the target censoring rate.

b: Not only the mean of exponential distribution was set, but the time when the study ends was set to 15 in order to attain the target censoring rate.

c: Not only the mean of exponential distribution was set, but the time when the study ends was set to 13 in order to attain the target censoring rate.

### Entry and follow-up times

The subjects were assumed to enter the study at different times during three time units periods. The entry times greater than 0 were generated from a uniform distribution between times 0 and 3 units. The maximum follow-up time was 12 units, which represents what often occurs in real data. [21–22] The subjects who were censored at time 12 units represented the subjects who remained at the end of the follow-up period. Thus, all subjects were followed up for a period of time that ranged between 3 units to 12 units, depending on their entry time. The follow-up time was set to 12 units, with the exception of the situations described in Table 2.

### Application of the methods

Considering all possible parameter selections, we ended up with several scenarios: 18 scenarios for the Weibull distribution and 12 scenarios for the lognormal distribution (Table 3).

**Table 3:** Summary of simulation variables.

Variable	Scenarios	Details
Sample size	2	100 or 500
		Weibull:
		γ = 0.6, λ = 6.65
		γ = 1, λ = 10
Parametric distribution	2	γ = 2, λ = 11.28
		lognormal:
		σ = 1, μ = 1.802585
		σ = 2, μ = 0.3025851
Censoring rate	3	0.76, 0.42 or 0.26

Although the sample size 100–500 covered the range of trials typically encountered well and reflect what was often seen in large trials, [16–18] additional work was performed by simulating lager trials (sample size set to 1000). The simulation were run with γ set to 0.6 and λ set to 6.65 and censoring rate set to 0.76 for Weibull distribution and σ set to 2 and μ set to 0.30 and censoring rate set to 0.76 for lognormal distribution.

For the Guyot et al. method, the number of events was used; however, this information is often not provided. For the Hoyle and Henley method, 6 time intervals were used in most cases.

For each scenario, 1000 datasets were generated. The four methods previously described were subsequently applied to each of the 1000 datasets, and the MLE was applied directly to the simulated IPD to estimate the parameters of the survival distribution, which commonly occurs in cost-effectiveness analyses. For each method, the mean survival time was estimated for each of the 1000 datasets. All simulations were conducted using the R for Statistical Computing version 3.1.1 (R Foundation for Statistical Computing, Vienna, Austria).

### Performance evaluation

The measurements used to evaluate the performance of the different methods have been summarized by Burton et al. [23]

Bias was calculated as follows:

$$\delta =\bar{\hat{\beta }}-\beta$$

in which β is the true value for the estimate of interest, and $\bar{\hat{\beta }}$ is the mean estimate of interest over the 1000 simulations performed.

Mean square error (MSE)

MSE is considered a useful measure of the overall accuracy because it incorporates both measures of bias and variability. The MSE was calculated as follows:

$$MSE={\left(\bar{\hat{\beta }}-\beta \right)}^{2}-{\left(SE(\hat{\beta })\right)}^{2}$$

in which $SE(\hat{\beta })$ was the empirical SE of the estimate of interest over all simulations.

### Uncertainty

In CEAs, the measure of effectiveness is the mean survival time over the duration of interest. [24] Thus, consideration of the uncertainty regarding the mean survival time is essential to conduct CEAs. [3] One of the advantages of using the methods proposed by Guyot et al. and Hoyle and Henley to conduct a CEA based on a published KM curve is that these methods can capture uncertainty regarding the mean survival time, unlike traditional methods, which are unable to reconstruct IPD.

To assess the performance of capturing the uncertainty reported by the two proposed methods, the standard errors of the mean survival time estimated by directly applying the MLE to actual IPD and by the two proposed methods were estimated.

We analyzed the impact of uncertainty on the estimate of mean time by applying a bootstrap resampling technique on the parameters of the survival distributions (Weibull and lognormal distributions). For every 1000 simulations, the standard deviation was estimated by applying a bootstrap resampling technique. For each bootstrap resample, the correlated variables were generated by applying the formula x = y+Tz, where x is the vector of the correlated variables, z is the vector of the independent standard normal variables, y is the vector of the parameter mean values and T is a cholesky decomposition of a variance-covariance matrix that was recorded from the simulations. The parameters of the survival distribution were acquired from the distribution, and the mean survival times were estimated. This three-step procedure was then repeated 10,000 times. The standard deviation of the mean survival time over the 10,000 bootstrap simulations was estimated. Thus, the standard error of the mean survival time for every 1000 simulations was obtained.

In the bootstrap simulations, uncertainty was captured as follows: Weibull distribution: (1) γ = 0.6, censoring rate = 0.76; (2) γ = 1, censoring rate = 0.76; (3) γ = 1, censoring rate = 0.26, and lognormal distribution: (4) σ = 2, censoring rate = 0.26.

## Results

For the tables and figures in this section, the methods are referred to as follows: the MLE was applied to the actual IPD (referred to as Actual IPD), the method proposed by Guyot et al. (referred to as Guyot et al.), the method proposed by Hoyle and Henley (referred to as Hoyle and Henley), the least squares method and the graphical method.

### Simulation results

**Fig 1:**
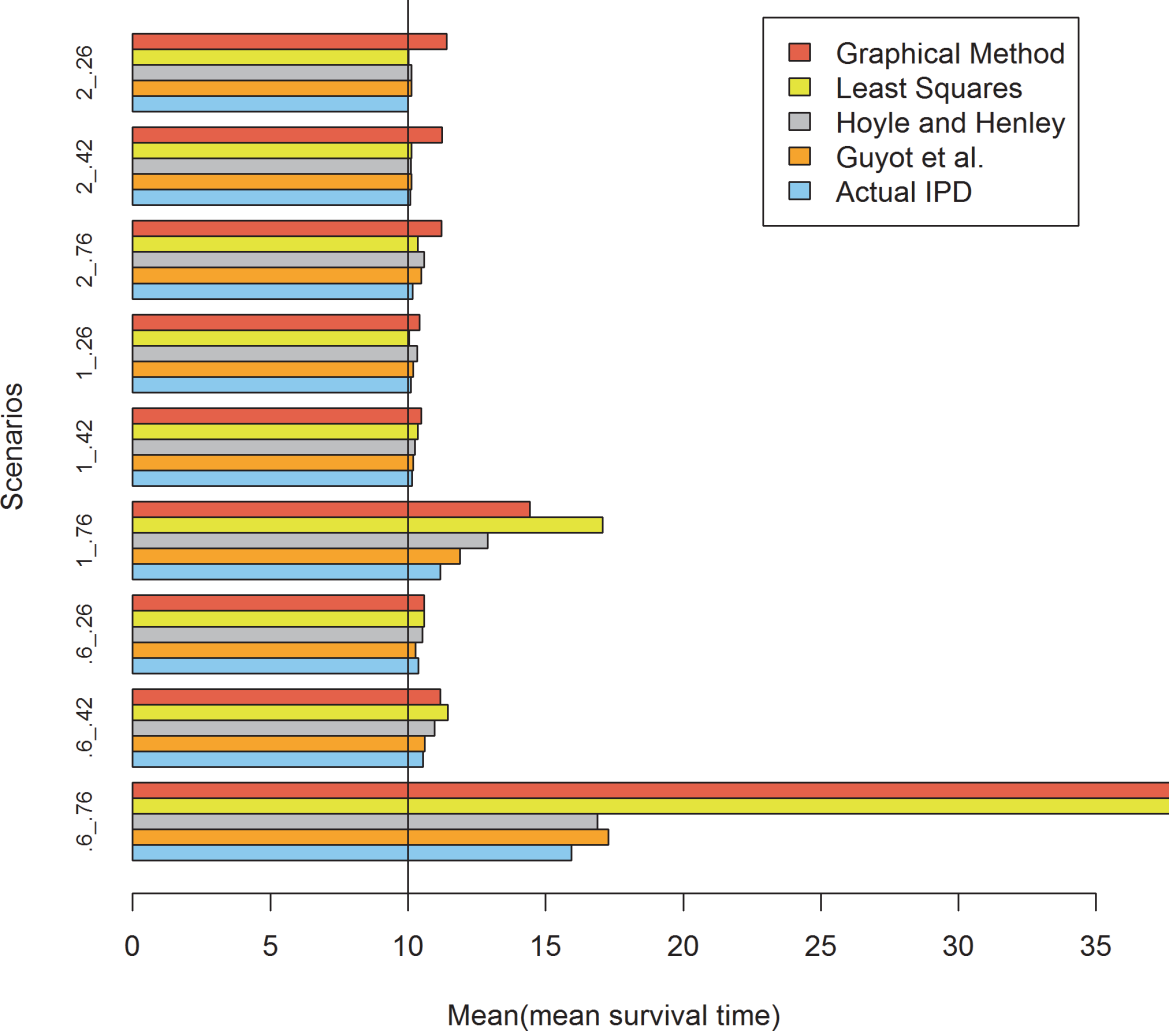
Simulation results for the five methods: 100 patients per trial. The values of .6, 1 and 2 represent the shape values of the Weibull distribution and .76, .42 and .26 represent the censoring rates.

Table 4 and Fig. 1 presents the results when the survival time followed a Weibull distribution and the sample size was 100. When the censoring rate was as low as 0.26, all four methods performed well in the estimation of the mean survival time, and their estimates were as accurate as the values estimated using actual IPD.

**Table 4:** Simulation results of mean survival time, bias, and MSE at 0.26, 0.42 and 0.76 censoring rate with sample size = 100 from three shapes of hazard function (weibull distribution).

Weibull parameter (γ,λ)	Censoring rate	Method	Mean survival time	Bias	MSE
0.6, 6.65	0.76	Actual IPD	15.9336	5.9341	37.1899
		Guyot et al.	17.2754	7.2760	53.9205
		Hoyle and Henley	16.8712	6.8718	47.6772
		Least Squares	3416	3406	15462811
		Graphical Method	357.54	347.54	160506
	0.42	Actual IPD	10.5470	0.5476	0.3125
		Guyot et al.	10.6075	0.6081	0.3834
		Hoyle and Henley	10.9734	0.9740	0.9687
		Least Squares	11.4388	1.4394	2.3522
		Graphical Method	11.1826	1.1832	1.5023
	0.26	Actual IPD	10.3671	0.3677	0.1432
		Guyot et al.	10.2806	0.2812	0.0869
		Hoyle and Henley	10.5170	0.5176	0.2769
		Least Squares	10.5891	0.5897	0.3611
		Graphical Method	10.5918	0.5924	0.3640
1, 10	0.76	Actual IPD	11.1790	1.1789	1.4209
		Guyot et al.	11.8904	1.8904	3.6299
		Hoyle and Henley	12.9022	2.9022	8.5909
		Least Squares	17.0620	7.0619	62.0269
		Graphical Method	14.4269	4.4269	22.7738
	0.42	Actual IPD	10.1433	0.1433	0.0239
		Guyot et al.	10.1801	0.1801	0.0364
		Hoyle and Henley	10.2534	0.2534	0.0700
		Least Squares	10.3630	0.3630	0.1572
		Graphical Method	10.4729	0.4729	0.2463
	0.26	Actual IPD	10.0939	0.0939	0.0107
		Guyot et al.	10.1793	0.1793	0.0349
		Hoyle and Henley	10.3346	0.3346	0.1150
		Least Squares	10.0475	0.0475	0.0056
		Graphical Method	10.4213	0.4213	0.1831
2, 11.28	0.76	Actual IPD	10.1699	0.1733	0.0319
		Guyot et al.	10.4740	0.4774	0.2334
		Hoyle and Henley	10.5882	0.5915	0.3555
		Least Squares	10.3604	0.3637	0.1397
		Graphical Method	11.2220	1.2254	1.5166
	0.42	Actual IPD	10.0818	0.0852	0.0078
		Guyot et al.	10.1202	0.1236	0.0160
		Hoyle and Henley	10.1014	0.1048	0.0117
		Least Squares	10.1346	0.1380	0.0199
		Graphical Method	11.2330	1.2363	1.5351
	0.26	Actual IPD	10.0060	0.0094	0.0004
		Guyot et al.	10.1296	0.1329	0.0182
		Hoyle and Henley	10.1312	0.1346	0.0187
		Least Squares	10.0244	0.0278	0.0015
		Graphical Method	11.4072	1.4105	1.9993

When the censoring rate was 0.42, all four methods provided satisfactory estimates of the mean survival time, with the least squares and graphical methods exhibiting small biases in the scenarios with a decreasing hazard rate.

When the censoring rate increased to 0.76, the least squares and graphical methods noticeably overestimated the mean survival time in the situations in which the hazard rate decreased or was constant. Because of the bias created from the application of MLE to actual IPD, the Guyot et al. and Hoyle and Henley methods provided biased estimates; however, these methods were still similar to the estimates obtained using actual IPD compared with the biases that resulted from the least squares and graphical methods.

When the sample size was 100 and the data followed a Weibull distribution, the accuracy of the estimates of mean survival time was somewhat dependent on the shape of the hazard function and the level of censoring. For decreasing and constant hazard patterns, the biases observed were greater in scenarios with higher compared with lower censoring rates, particularly for a decreasing hazard pattern; these biases resulted from the use of MLE when the data were heavily censored. There may be a substantially greater amount of bias with decreasing compared with constant hazard rates. One potential explanation is that the bias magnitude from the MLE is more affected by decreasing compared with constant hazard rates.

When the censoring rate was the same, e.g., 0.76 or 0.42, the magnitude of the bias was affected by the shape of hazard function of the survival models. There was a greater bias with decreasing compared with increasing or constant hazard rates. When the shape parameter was equal to 0.6, which corresponds to a decreasing hazard function, the percentage bias in the Actual IPD, the method of Guyot et al. and the Hoyle and Henley method was 59.34, 72.76 and 68.72%, respectively, whereas the percentage bias was 11.79, 18.90 and 29.02%, respectively, with a constant hazard rate and 0.09, 1.33 and 1.35%, respectively, with an increasing hazard rate. One potential explanation is that the amount of random censoring in the increasing hazard case was less than the decreasing and constant hazard cases.

**Fig 2:**
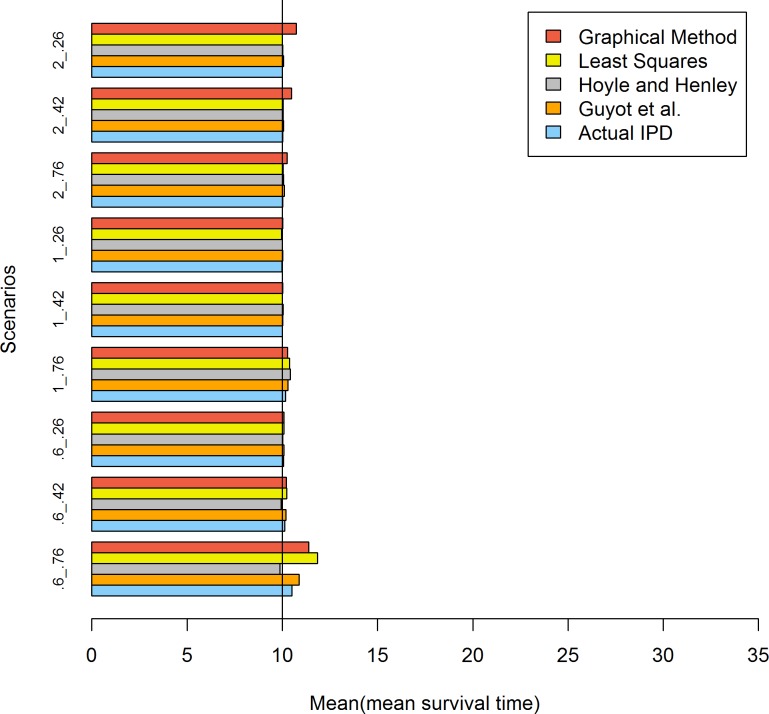
Simulation results for the five methods: 500 patients per trial. The values of .6, 1 and 2 represent the shape values of the Weibull distribution and .76, .42 and .26 represent the censoring rates.

The results from Table 5 and Fig. 2 demonstrate that all methods performed well when the sample size was 500.

**Table 5:** Simulation results of mean survival time, bias, and MSE at 0.26, 0.42 and 0.76 censoring rate with sample size = 500 from three shapes of hazard function (weibull distribution).

Weibull parameter (γ,λ)	Censoring rate	Method	Mean estimate	Bias	MSE
0.6, 6.65	0.76	Actual IPD	10.5027	0.5033	0.2645
		Guyot et al.	10.8878	0.8884	0.8038
		Hoyle and Henley	9.8664	-0.1331	0.0311
		Least Squares	11.8471	1.8477	3.4665
		Graphical Method	11.3832	1.3838	1.9476
	0.42	Actual IPD	10.1298	0.1304	0.0189
		Guyot et al.	10.1962	0.1968	0.0408
		Hoyle and Henley	9.9430	-0.0564	0.0055
		Least Squares	10.2278	0.2284	0.0554
		Graphical Method	10.2139	0.2145	0.0491
	0.26	Actual IPD	10.0585	0.0591	0.0048
		Guyot et al.	10.0899	0.0905	0.0095
		Hoyle and Henley	10.0123	0.0129	0.0015
		Least Squares	10.0802	0.0808	0.0082
		Graphical Method	10.0751	0.0757	0.0073
1, 10	0.76	Actual IPD	10.1764	0.1764	0.0330
		Guyot et al.	10.2965	0.2965	0.0906
		Hoyle and Henley	10.4190	0.4190	0.1785
		Least Squares	10.3785	0.3785	0.1477
		Graphical Method	10.2608	0.2608	0.0715
	0.42	Actual IPD	10.0050	0.0050	0.0005
		Guyot et al.	10.0226	0.0226	0.0011
		Hoyle and Henley	10.0366	0.0366	0.0019
		Least Squares	9.9990	-0.0010	0.0006
		Graphical Method	10.0165	0.0165	0.0010
	0.26	Actual IPD	9.9738	-0.0262	0.0010
		Guyot et al.	10.0195	0.0195	0.0008
		Hoyle and Henley	10.0079	0.0079	0.0005
		Least Squares	9.9587	-0.0413	0.0023
		Graphical Method	10.0106	0.0106	0.0007
2, 11.28	0.76	Actual IPD	10.0202	0.0236	0.0009
		Guyot et al.	10.1055	0.1088	0.0124
		Hoyle and Henley	10.0576	0.0609	0.0042
		Least Squares	10.0348	0.0381	0.0020
		Graphical Method	10.2487	0.2521	0.0648
	0.42	Actual IPD	10.0291	0.0325	0.0012
		Guyot et al.	10.0634	0.0668	0.0046
		Hoyle and Henley	10.0321	0.0354	0.0014
		Least Squares	10.0366	0.0400	0.0017
		Graphical Method	10.4739	0.4772	0.2287
	0.26	Actual IPD	9.9996	0.0030	0.0001
		Guyot et al.	10.0689	0.0722	0.0053
		Hoyle and Henley	10.0232	0.0266	0.0008
		Least Squares	9.9957	-0.0010	0.0001
		Graphical Method	10.7278	0.7312	0.5361

**Fig 3:**
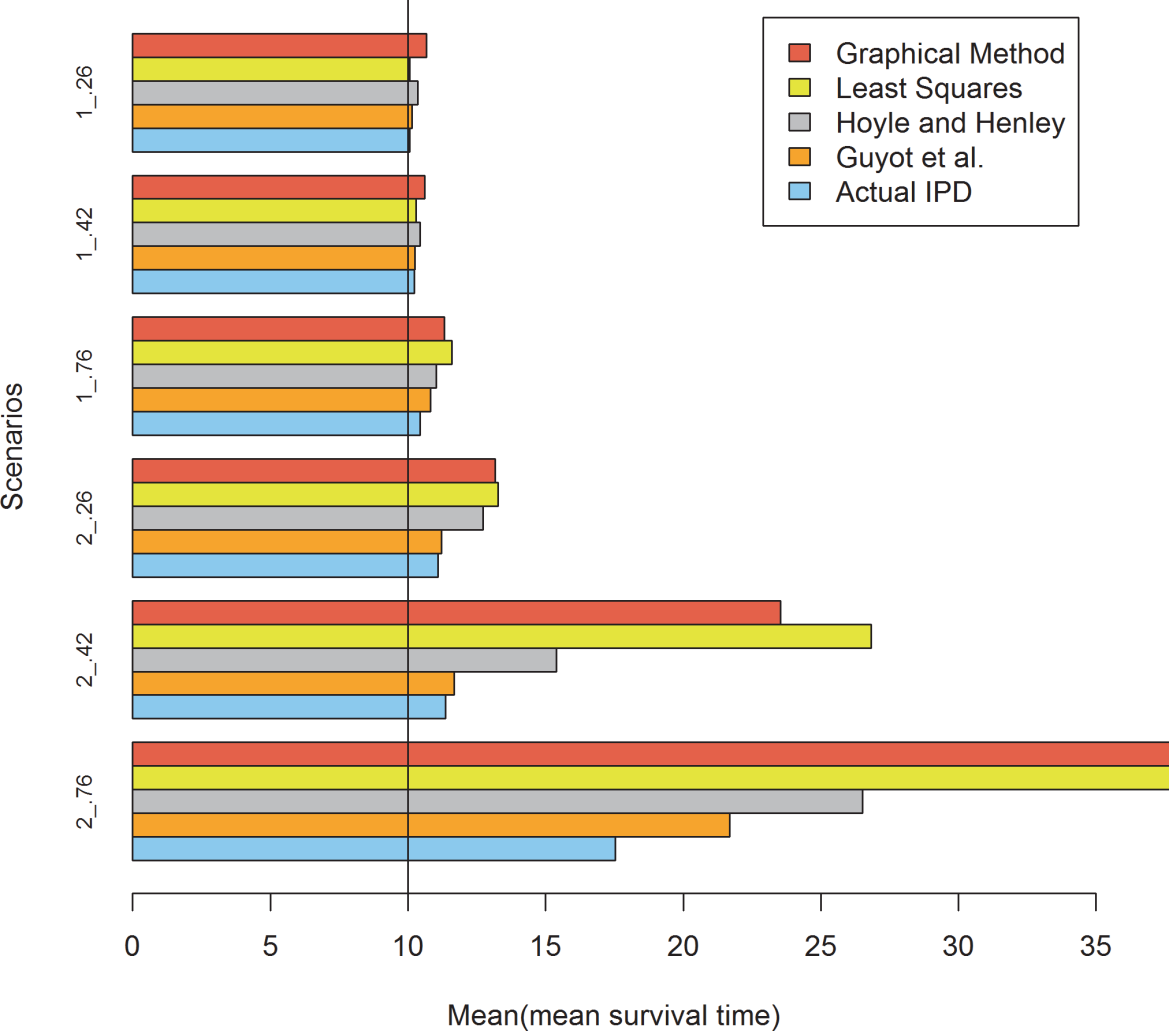
Simulation results for the five methods: 100 patients per trial. The values of 2 and 1 represent the sigma values of the lognormal distribution and .76, .42 and .26 represent the censoring rates.

Table 6 and Fig. 3 presents the results when the survival time followed a lognormal distribution and the sample size was 100. When σ was 1 and μ was 1.80, all methods performed well at all three levels of censoring. When σ was 2 and μ was 0.30, the magnitude of the bias was strongly affected by the level of censoring. The greater the degree of censoring, the larger the bias. The least squares and graphical methods clearly overestimated the estimation of the mean survival time. As a result of the MLE bias, the Guyot et al. and Hoyle and Henley methods also overestimated the estimates. The Guyot et al. method performed noticeably better than the Hoyle and Henley method when σ = 2 and μ = 0.30 and the censoring rates = 0.76 and 0.42.

**Table 6:** Simulation results of mean survival time, bias, and MSE at 0.26, 0.42 and 0.76 censoring rate with sample size = 100 from two shapes of hazard function (lognormal distribution).

lognormal parameter (σ, μ)	Censoring rate	Method	Mean estimate	Bias	MSE
1, 1.80	0.76	Actual IPD	10.4327	0.4327	0.2016
		Guyot et al.	10.8130	0.8130	0.6828
		Hoyle and Henley	11.0261	1.0261	1.0759
		Least Squares	11.6033	1.6033	2.6679
		Graphical Method	11.3256	1.3256	1.8004
	0.42	Actual IPD	10.2251	0.2251	0.0543
		Guyot et al.	10.2424	0.2424	0.0627
		Hoyle and Henley	10.4322	0.4322	0.1911
		Least Squares	10.2969	0.2969	0.0944
		Graphical Method	10.6083	0.6083	0.3757
	0.26	Actual IPD	10.0651	0.0651	0.0064
		Guyot et al.	10.1453	0.1453	0.0238
		Hoyle and Henley	10.3488	0.3488	0.1246
		Least Squares	10.0612	0.0612	0.0073
		Graphical Method	10.6728	0.6728	0.4574
2, 0.30	0.76	Actual IPD	17.5418	7.5418	58.1389
		Guyot et al.	21.6860	11.6860	139.4465
		Hoyle and Henley	26.5135	16.5135	274.3040
		Least Squares	6936	6926	90206329
		Graphical Method	221.712	211.712	55210
	0.42	Actual IPD	11.3590	1.3590	1.9006
		Guyot et al.	11.6869	1.6869	2.9204
		Hoyle and Henley	15.3891	5.3891	29.3930
		Least Squares	26.8246	16.8246	415.537
		Graphical Method	23.5345	13.5345	267.124
	0.26	Actual IPD	11.0847	1.0847	1.2089
		Guyot et al.	11.2215	1.2215	1.5269
		Hoyle and Henley	12.7159	2.7159	7.5270
		Least Squares	13.2615	3.2615	10.9698
		Graphical Method	13.1601	3.1601	10.3016

**Fig 4:**
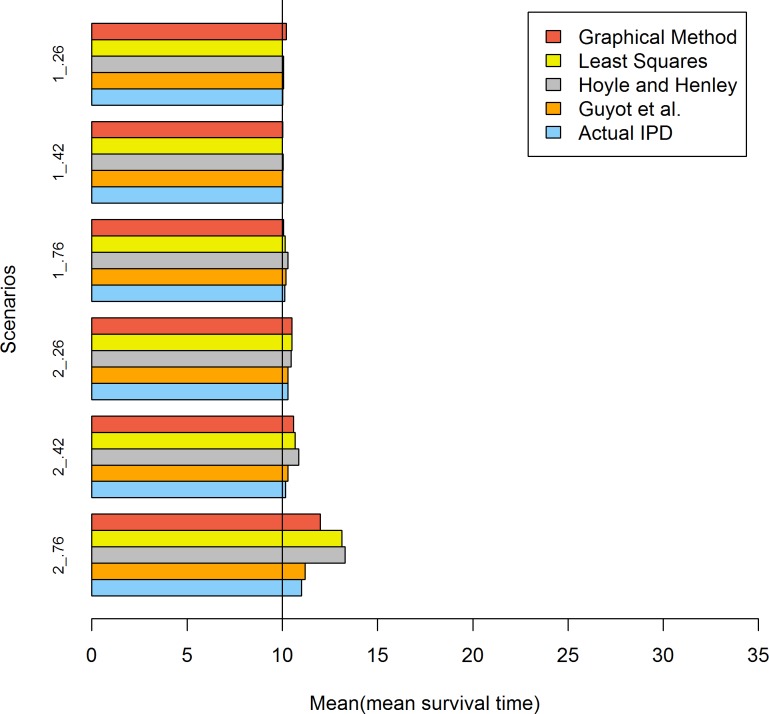
Simulation results for the five methods: 500 patients per trial. The values of 2 and 1 represent the sigma values of the lognormal distribution and .76, .42 and .26 represent the censoring rates.

Table 7 and Fig. 4 presents the results when the survival time followed a lognormal distribution and the sample size was 500. When σ was 1 and μ was 1.80, all methods performed well. When σ was 2 and μ was 0.30, a greater bias was observed in the Hoyle and Henley method.

**Table 7:** Simulation results of mean survival time, bias, and MSE at 0.26, 0.42 and 0.76 censoring rate with sample size = 500 from two shapes of hazard function (lognormal distribution).

lognormal parameter (σ, μ)	Censoring rate	Method	Mean estimate	Bias	MSE
1, 1.80	0.76	Actual IPD	10.1337	0.1337	0.0197
		Guyot et al.	10.1941	0.1941	0.0399
		Hoyle and Henley	10.2861	0.2861	0.0842
		Least Squares	10.1359	0.1359	0.0216
		Graphical Method	10.0661	0.0661	0.0067
	0.42	Actual IPD	10.0124	0.0124	0.0007
		Guyot et al.	10.0180	0.0180	0.0009
		Hoyle and Henley	10.0434	0.0434	0.0025
		Least Squares	10.0054	0.0054	0.0008
		Graphical Method	10.0239	0.0239	0.0013
	0.26	Actual IPD	10.0171	0.0171	0.0008
		Guyot et al.	10.0531	0.0531	0.0033
		Hoyle and Henley	10.0543	0.0543	0.0035
		Least Squares	9.9953	-0.0047	0.0006
		Graphical Method	10.2133	0.2133	0.0462
2, 0.30	0.76	Actual IPD	11.0017	1.0017	1.0305
		Guyot et al.	11.1888	1.1888	1.4487
		Hoyle and Henley	13.3028	3.3028	10.9742
		Least Squares	13.1146	3.1146	9.8380
		Graphical Method	11.9960	1.9960	4.0446
	0.42	Actual IPD	10.1653	0.1653	0.0329
		Guyot et al.	10.2860	0.2860	0.0881
		Hoyle and Henley	10.8518	0.8518	0.7367
		Least Squares	10.6640	0.6640	0.4567
		Graphical Method	10.5780	0.5780	0.3479
	0.26	Actual IPD	10.2829	0.2829	0.0846
		Guyot et al.	10.3012	0.3011	0.0955
		Hoyle and Henley	10.4534	0.4534	0.2142
		Least Squares	10.5075	0.5075	0.2675
		Graphical Method	10.4920	0.4920	0.2520

The results demonstrated that the biases were also affected by the shape of the hazard function and the level of censoring in both the lognormal and Weibull distributions, regardless of the sample size. For the same high censoring rate, a greater bias will be produced in the decreasing hazard pattern compared with the other hazard patterns.

**Fig 5:**
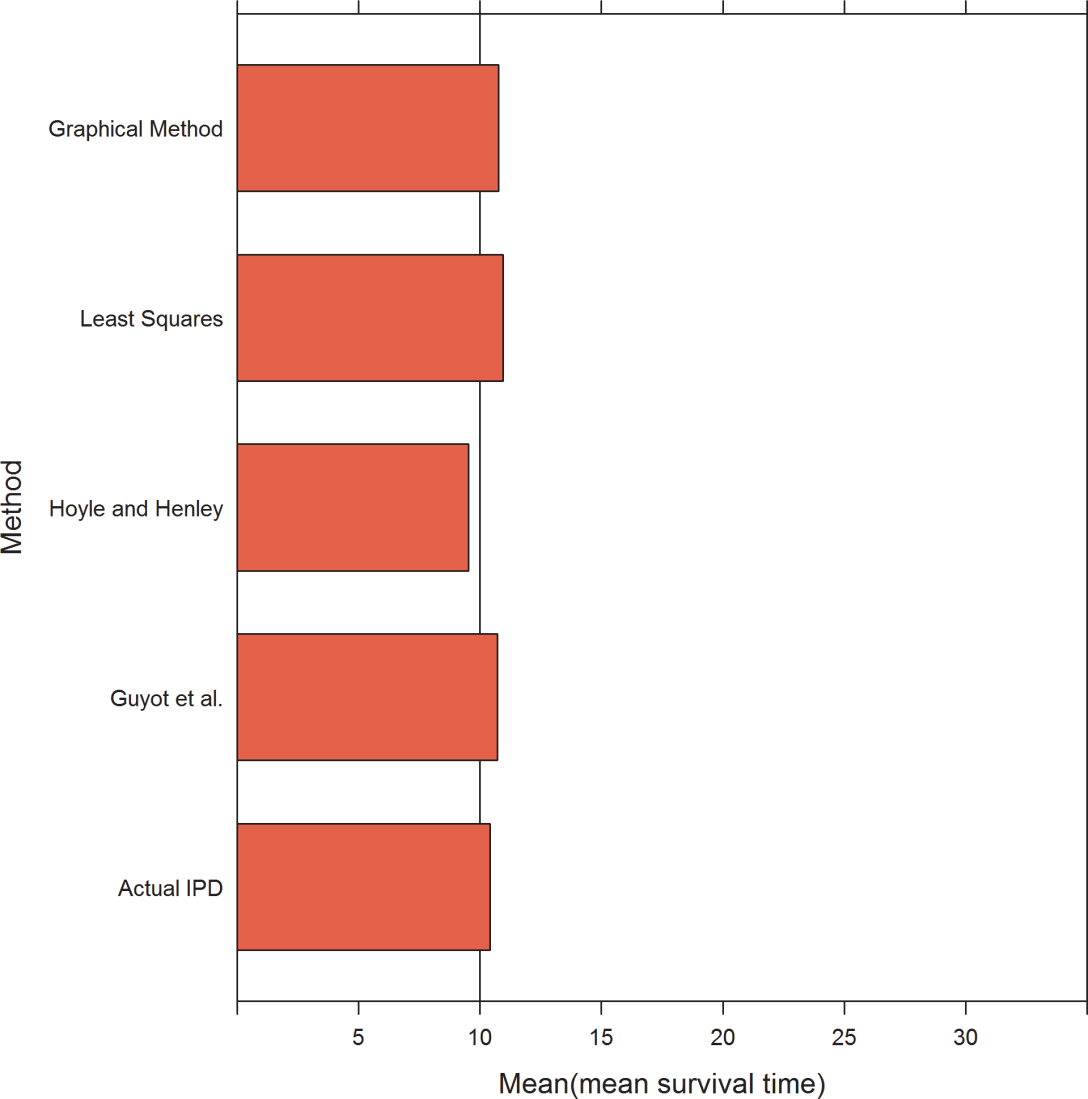
Simulation results for the five methods: 1000 patients per trial. The simulation were run with γ set to 0.6 and λ set to 6.65 and censoring rate set to 0.76 for Weibull distribution.

**Fig 6:**
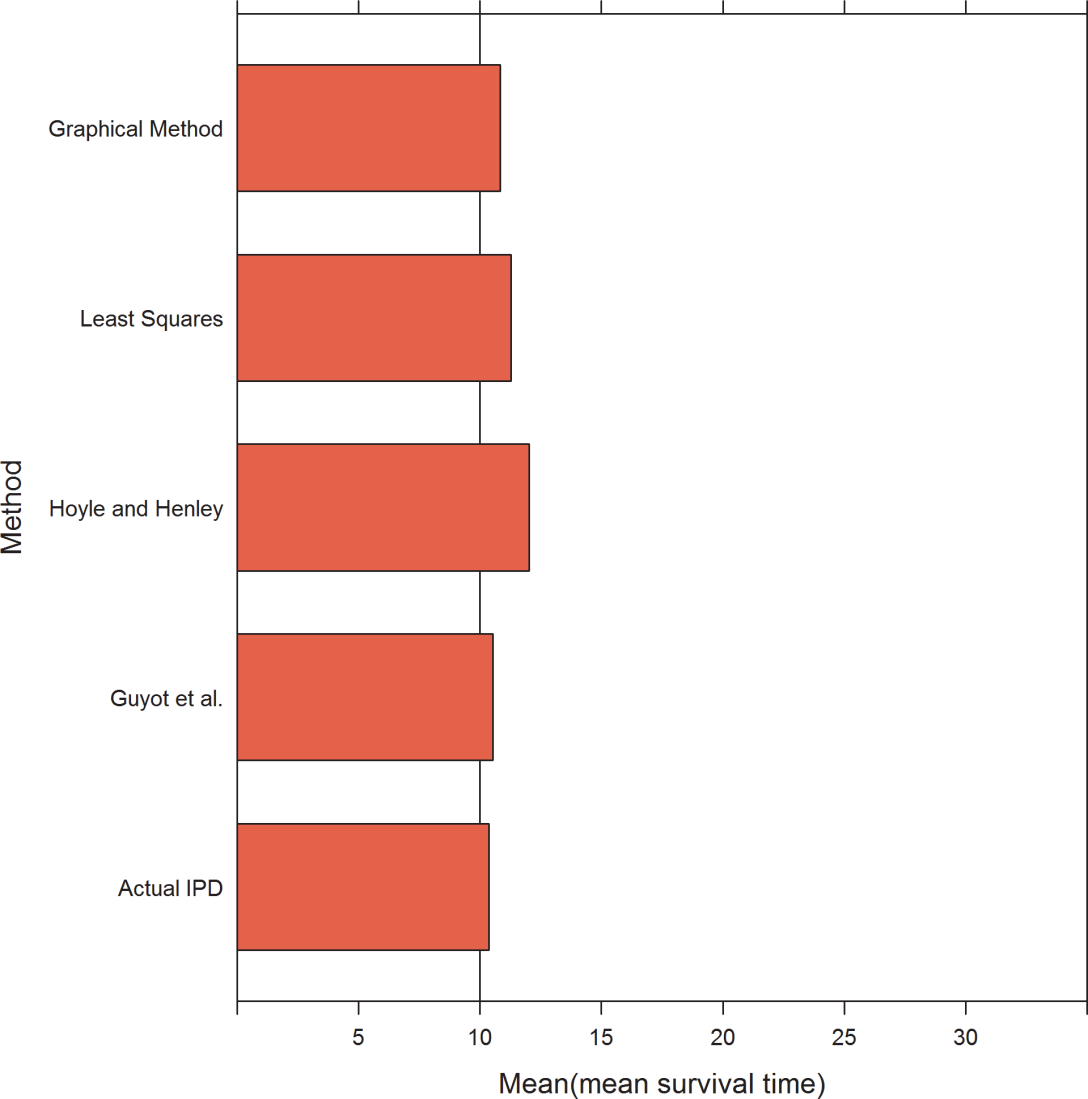
Simulation results for the five methods: 1000 patients per trial. The simulation were run with σ set to 2 and μ set to 0.30 and censoring rate set to 0.76 for lognormal distribution.

The Fig. 5 and Fig. 6 presents the results when sample size was 1000 and survival time followed a Weibull distribution and lognormal distribution, respectively. The results showed that the performances of all methods in the cases of the larger trial were consistent with those in the cases of the sample size 500.

### Uncertainty results

**Fig 7:**
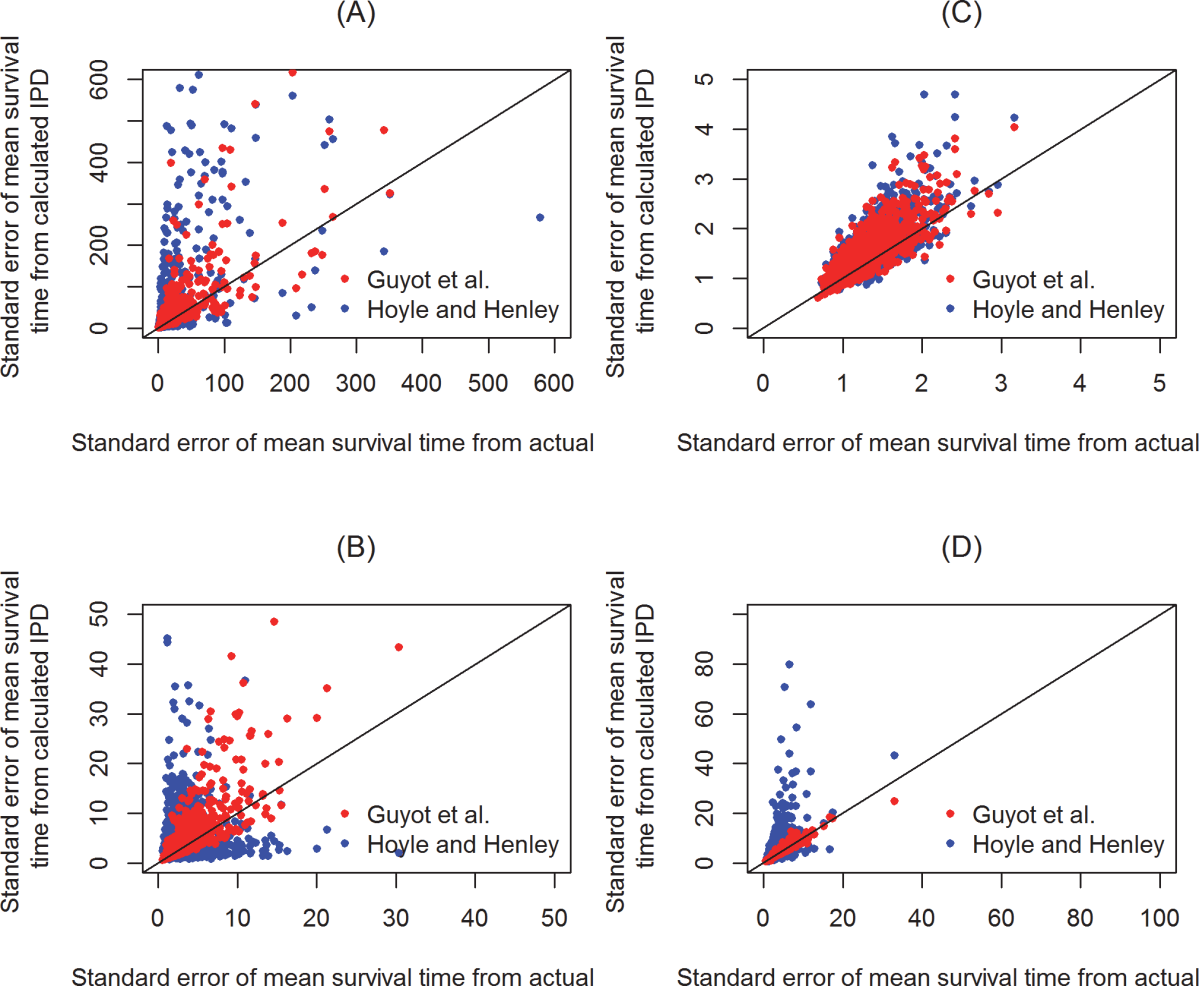
The results of uncertainty regarding the estimate of mean survival time. The uncertainty was captured in the following situations: Weibull distribution: A. γ = 0.6, censoring rate = 0.76; B. γ = 1, censoring rate = 0.76; C. γ = 1, censoring rate = 0.26, and lognormal distribution: D. σ = 2, censoring rate = 0.26.

The results of Fig. 7 indicate that in situations that use the Weibull distribution, the Guyot et al. method provided slightly better uncertainty measures of the mean survival time than the Hoyle and Henley method. When the lognormal distribution was used, the mean difference between the standard error of the mean survival time from actual IPD and the corresponding standard error reported by the Guyot et al. method was substantially less than the Hoyle and Henley method.

## Discussion

As expected, the application of the MLE to actual IPD was the most accurate method compared with the other methods in the simulation study; however, it provided noticeable biases in situations in which the level of censoring was high and the hazard function decreased over time. The biases occurred because the MLE can be quite biased when the sample size is small or when the survival data are heavily censored. [25–26] To correct this problem, a modified MLE (MMLE) was proposed to reduce bias in the estimates of the Weibull shape parameter through the modification of the profile likelihood [26]. The MLE and MMLE work well for complete, Type I, or Type II censored data, but they are not useful for random censored data. More recently, Shen [10] proposed a method that can be applied not only to censored data as previously discussed but also to other data, such as random censoring, progressive Type II censoring, and adaptive Type II progressive censoring. If the MMLE or the method proposed by Shen is applied to IPD, the bias will be reduced. However, the most commonly used statistical software packages do not include programs for MMLE or the method proposed by Shen. This debate is beyond our study’s scope, and we believe that bias will be reduced as long as the programs for the corrected-MLE method are included in statistical software.

In general, the least squares and graphic methods provided biased estimates; the estimates were heavily biased in some scenarios. For the Weibull distribution, the bias differences between the two proposed methods were not significant. Therefore, both of the two proposed methods provided satisfactory estimates of the mean survival time; these estimates were almost as accurate as the estimates obtained using actual IPD. However, the number of events was used in the simulation for the Guyot et al. method, and this information is often not provided in clinical trials. Therefore, the simulation design favored the Guyot et al. method. In addition, the Guyot et al. method required substantially more data preparation work compared with the Hoyle and Henley method. The data prepared for the Guyot et al. method should be sufficient, and every step in the KM curves should be captured. Hundreds of data points were required for the Guyot et al. method, whereas the number of data points required for the Hoyle and Henley method was substantially less; for example, when 6 time points were reported, 24 data points were required for Hoyle and Henley method. There is a larger probability for bias to occur from data extraction when it is necessary to extract more data. In the simulation study, the actual survival probabilities were used instead of the probabilities obtained from the published survival curves. Therefore, the results from the simulation study were the most accurate results that could be obtained from the four methods. Six time intervals were used for most scenarios in the simulation. The Hoyle and Henley method worked better when more time intervals were reported in the clinical trial. Published data typically include a minimum of 6 time intervals. Therefore, the accuracy of the Hoyle and Henley method assessed here is likely the minimum accuracy that can be obtained from this method.

Given that both the Guyot et al. and Hoyle and Henley methods provided satisfactory estimates of the mean survival time when the Weibull distribution was fitted, we believe that the Hoyle and Henley method would not perform worse than the Guyot et al. method in the real world. For the lognormal distribution, the Hoyle and Henley method resulted in more noticeable biases compared with the other methods potentially because the Hoyle and Henley method is affected to a greater degree by the long tail of the lognormal distribution.

In the simulation, the sample sizes of 100 and 500 were chosen, because the sample size 100–500 covers the range of trials typically encountered well and reflect what is often seen in large trials. [16–18] However, there are trials in which sample size is less than 100 or more than 500. The results showed that the performances of all methods in the cases of the larger trial are consistent with those in the cases of the sample size 500. For small trials, it’s possible to see the exact drops in the Kaplan Meier curve and the tick marks for censorships. In these situations we don’t need to use the two proposed methods.

## Conclusions

The objective of this article was to review and compare the methods used to recreate individual patient data for economic evaluations from published Kaplan-Meier survival curves using a Monte Carlo simulation. Of the methods assessed in this study, the least squares and graphical methods were the least preferred methods because of their remarkable overestimation in some situations. Because the simulation design favored the Guyot et al. method and the accuracy of the Hoyle and Henley method assessed in the simulation is likely the minimum accuracy that can be obtained from this method, both the Hoyle and Henley and Guyot et al. methods provided satisfactory estimates and uncertainty values of the mean survival time for the Weibull distribution; these estimates were as accurate as the values estimated using actual IPD. If the lognormal distribution was utilized, the Guyot et al. method performed noticeably better when sigma equaled 2 and the censoring rate was high, e.g., 0.76 and 0.42, compared with the Hoyle and Henley method.
